# Active Site Mutations as a Suitable Tool Contributing to Explain a Mechanism of Aristolochic Acid I Nitroreduction by Cytochromes P450 1A1, 1A2 and 1B1

**DOI:** 10.3390/ijms17020213

**Published:** 2016-02-05

**Authors:** Jan Milichovský, František Bárta, Heinz H. Schmeiser, Volker M. Arlt, Eva Frei, Marie Stiborová, Václav Martínek

**Affiliations:** 1 Department of Biochemistry, Faculty of Science, Charles University, Albertov 2030, CZ-12843 Prague 2, Czech Republic; jan.milichovsky@natur.cuni.cz (J.M.); frantisek.barta@natur.cuni.cz (F.B.); evafrei@t-online.de (E.F.); 2 Division of Radiopharmaceutical Chemistry, German Cancer Research Center (DKFZ), Im Neuenheimer Feld 280, 69120 Heidelberg, Germany; h.schmeiser@dkfz-heidelberg.de; 3 Analytical and Environmental Sciences Division, MRC-PHE Centre for Environment and Health, King’s College London, London SE1 9NH, UK; volker.arlt@kcl.ac.uk

**Keywords:** aristolochic acid nephropathy, aristolochic acid I, site-directed mutagenesis of cytochromes P450 1A1, 1A2 and 1B1, nitroreduction, DNA adduct formation

## Abstract

Aristolochic acid I (AAI) is a plant drug found in *Aristolochia* species that causes aristolochic acid nephropathy, Balkan endemic nephropathy and their associated urothelial malignancies. AAI is activated via nitroreduction producing genotoxic *N*-hydroxyaristolactam, which forms DNA adducts. The major enzymes responsible for the reductive bioactivation of AAI are NAD(P)H:quinone oxidoreductase and cytochromes P450 (CYP) 1A1 and 1A2. Using site-directed mutagenesis we investigated the possible mechanisms of CYP1A1/1A2/1B1-catalyzed AAI nitroreduction. Molecular modelling predicted that the hydroxyl groups of serine122/threonine124 (Ser122/Thr124) amino acids in the CYP1A1/1A2-AAI binary complexes located near to the nitro group of AAI, are mechanistically important as they provide the proton required for the stepwise reduction reaction. In contrast, the closely related CYP1B1 with no hydroxyl group containing residues in its active site is ineffective in catalyzing AAI nitroreduction. In order to construct an experimental model, mutant forms of CYP1A1 and 1A2 were prepared, where Ser122 and Thr124 were replaced by Ala (CYP1A1-S122A) and Val (CYP1A2-T124V), respectively. Similarly, a CYP1B1 mutant was prepared in which Ala133 was replaced by Ser (CYP1B1-A133S). Site-directed mutagenesis was performed using a quickchange approach. Wild and mutated forms of these enzymes were heterologously expressed in *Escherichia coli* and isolated enzymes characterized using UV-vis spectroscopy to verify correct protein folding. Their catalytic activity was confirmed with CYP1A1, 1A2 and 1B1 marker substrates. Using ^32^P-postlabelling we determined the efficiency of wild-type and mutant forms of CYP1A1, 1A2, and 1B1 reconstituted with NADPH:CYP oxidoreductase to bioactivate AAI to reactive intermediates forming covalent DNA adducts. The S122A and T124V mutations in CYP1A1 and 1A2, respectively, abolished the efficiency of CYP1A1 and 1A2 enzymes to generate AAI-DNA adducts. In contrast, the formation of AAI-DNA adducts was catalyzed by CYP1B1 with the A133S mutation. Our experimental model confirms the importance of the hydroxyl group possessing amino acids in the active center of CYP1A1 and 1A2 for AAI nitroreduction.

## 1. Introduction

**Figure 1:**
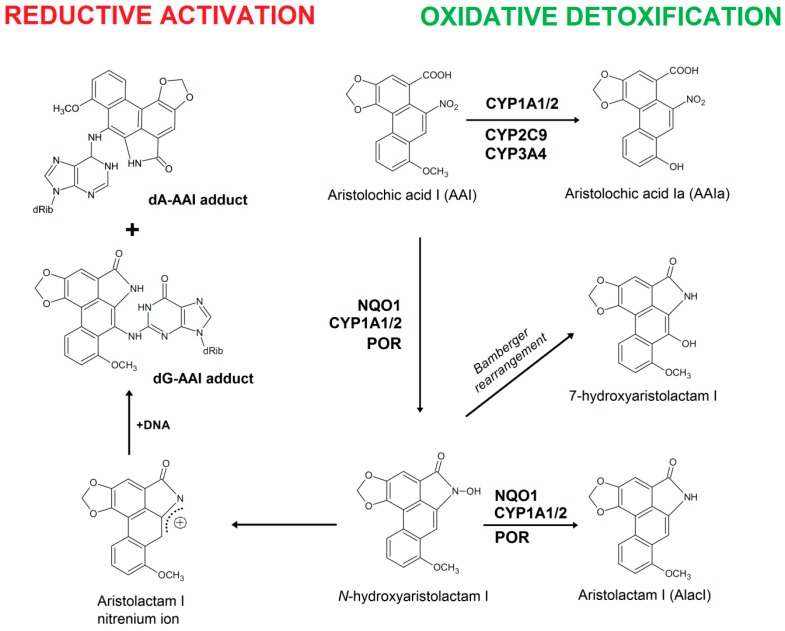
Activation and detoxification pathways of AAI. dA-AAI, 7-(deoxyadenosin-*N*^6^-yl)aristolactam I; dG-AAI, 7-(deoxyguanosin-*N*^2^-yl)aristolactam I; CYP1A1/2, cytochrome P450 1A1 and 1A2; CYP2C9, cytochrome P450 2C9; CYP3A4, cytochrome P450 3A4; NQO1, NAD(P)H:quinone oxidoreductase.

The plant drug aristolochic acid (AA) found in *Aristolochia* species has been shown to cause the so-called Chinese herbs nephropathy, now termed aristolochic acid nephropathy (AAN) [1,2]. The unique renal fibrosis is associated with development of upper urothelial tract carcinoma (UUC) [3,4,5,6]. As described by the International Agency for Research on Cancer, AA belongs to a Group I carcinogen [7]. Dietary exposure to AA has also been associated with Balkan endemic nephropathy (BEN) and urothelial cancer [8,9]; this nephropathy is endemic in several rural areas of Serbia, Bosnia, Croatia, Bulgaria and Romania [10]. The plant extract AA is a mixture of structurally related nitrophenanthrene carboxylic acids, the major components being aristolochic acid I (AAI) (see Figure 1) and aristolochic acid II (AAII).

AAI is considered to be the major cause of AAN-associated malignancy (*i.e.*, UUC development) and in order to exert its carcinogenic properties metabolic activation is required. In contrast, AAI is thought to directly mediate interstitial nephropathy [8,9,11,12,13,14,15]. The reductive activation of the nitro group of AAI results in generation of *N*-hydroxyaristolactam I that subsequently decomposes to a cyclic acylnitrenium ion forming DNA adducts (Figure 1) [2]. Specific AAI-DNA adducts were found in kidney and other organs of patients suffering from AAN and BEN and are used as biomarkers of AA exposure [9,10,16,17,18]. 7-(Deoxyadenosin-*N*^6^-yl)aristolactam I (dA-AAI) is the most abundant DNA adduct formed, exhibiting extremely long persistence in urothelial DNA [1,5,10,18,19]. This adduct generates characteristic A to T transversion mutations in *TP53* in urothelial tumours of AAN and BEN patients and experimental systems [8,9,20,21]. This process is considered to be an important molecular mechanism of AA-derived carcinogenesis [8,22]. The A–T mutations have also been found in other loci by whole-genome and exome sequencing after AA exposure [23,24,25,26]. 

Reduction of AAI is catalyzed by both cytosolic and microsomal enzymes; in this reaction NAD(P)H:quinone oxidoreductase (NQO1) is one of the most important cytosolic nitroreductases [12,13,27,28,29,30] (Figure 1). On the contrary, cytosolic conjugation enzymes such as human sulfotransferases (SULTs 1A1, 1A2, 1A3, 2E1 and 2A1) or *N*,*O*-acetyltransferases 1 and 2 were found not to be involved in AAI activation [31,32]. However, recently Grollman with his collaborators [33] have found that not only *N*-hydroxyaristolactam I alone forms AAI-DNA adducts. They showed that in the presence of human NQO1, the ability of AAI to form AAI-derived DNA adducts was significantly increased by adding the SULT1B1 with its cofactor, 3’-phosphoadenosine-5’-phosphosulfate. In human liver microsomes, AAI is most effectively bioactivated by cytochrome P450 (CYP) 1A2, followed by CYP1A1. NADPH:CYP oxidoreductase (POR) is able to reduce AAI too, but to a lower extent [34,35,36]. Of human recombinant CYP enzymes, CYP1A2 and CYP1A1 are the most effective to reductively activate AAI, while other CYPs are almost ineffective in this reaction [36,37,38]. Human CYP1A1 and 1A2 are also the major enzymes participating in oxidative detoxification of AAI to its *O*-demethylated product, 8-hydroxyaristolochic acid I (aristolochic acid Ia, AAIa) [39,40,41,42,43,44] (Figure 1). The highest impact on AAI detoxification in human liver is attributed to CYP1A2 (nearly 50%), followed by CYP2C9, CYP3A4, and CYP1A1 (~10%–15% each) [44].

Whereas the mechanism responsible for CYP-catalyzed oxidation reactions including oxidative *O*-demethylation of AAI has been partially explained [44,45], information on the mechanism of AAI nitroreduction catalyzed by these enzymes is still lacking. Previous studies using theoretical approaches such as molecular modeling, able to evaluate interactions of AAI with the active sites of human CYP1A1, 1A2 and 1B1 under the reductive conditions, have predicted that the hydroxyl groups of Ser122/Thr124 residues in the CYP1A1/1A2-AAI binary complexes are critical determinants of AAI nitroreduction [37,38]. These hydroxyl groups in the CYP1A1/1A2-AAI binary complexes are closely placed to the nitro group of the AAI, thereby being able to provide a proton necessary for the stepwise reduction reaction. In contrast, the closely related CYP1B1, which is lacking the hydroxyl group containing residues in its active site, is ineffective in catalyzing AAI nitroreductase activity [36,37,38]. 

In this study we used site-directed mutagenesis in an experimental expression system to further investigate the possible mechanisms of AAI nitroreduction catalyzed by CYP1A1, 1A2, and 1B1. Wild-type and mutant forms of human CYP1A1, 1A2 and 1B1 were prepared in a heterologous expression system of *Escherichia coli* and purified by immobilized-metal affinity chromatography [45]. In the mutant forms of CYP1A1, 1A2 and 1B1, Ser122, Thr124 and Ala134 were replaced by Ala122, Val124 and Ser134, respectively. The isolated enzymes were used to investigate their efficiencies to reductively activate AAI to reactive species forming DNA adducts using ^32^P-postlabelling.

## 2. Results and Discussion

### 2.1. Expression of Human Wild-Type and Mutant CYP1A1, 1A2, and 1B1 Enzymes in E. coli and Their Purification 

In this study human wild-type CYP1A1, 1A2 and 1B1 and mutant CYP1A1-S122A, CYP1A2-T124V, and CYP1B1-A133S enzymes were expressed in *E. coli* as N-terminally modified forms (see the Experimental Section) to achieve successful bacterial expression. 

The enzymes expressed in *E. coli* were isolated by immobilized-metal affinity chromatography essentially as described [46,47] with minor modification (see the Experimental Section). 

**Figure 2:**
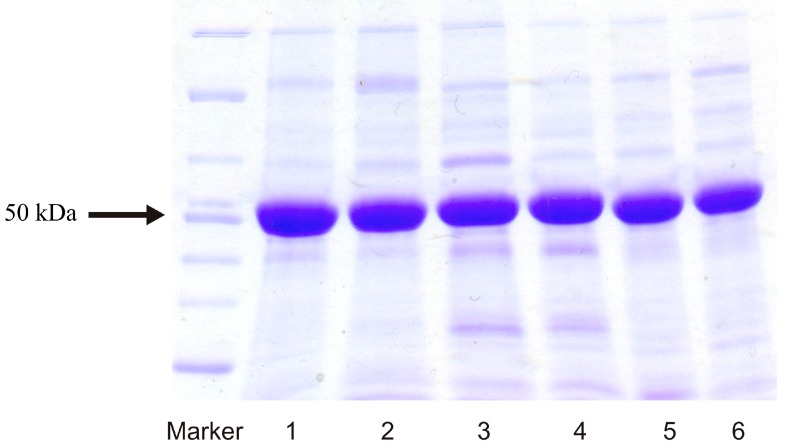
SDS-PAGE of final preparations of recombinant CYP enzymes. Two-hundred pmol of CYP1A1 (lane 1), CYP1A1-S122A (lane 2), CYP1A2 (lane 3), CYP1A2-T124V (lane 4), CYP1B1 (lane 5) and CYP1B1-A133S (lane 6) was loaded onto gradient 4%–20% gel. The arrow indicates a protein band having a molecular mass of ~50 kDa.

Using sodium dodecyl sulfate-polyacrylamide gel electrophoresis (SDS-PAGE), the purified CYP enzymes were shown to represent more than 97% of the enzyme proteins containing a low amount of impurities of other proteins (Figure 2). The isolated CYPs have a molecular mass of ~50 kDa (see the arrow in Figure 2). 

**Figure 3:**
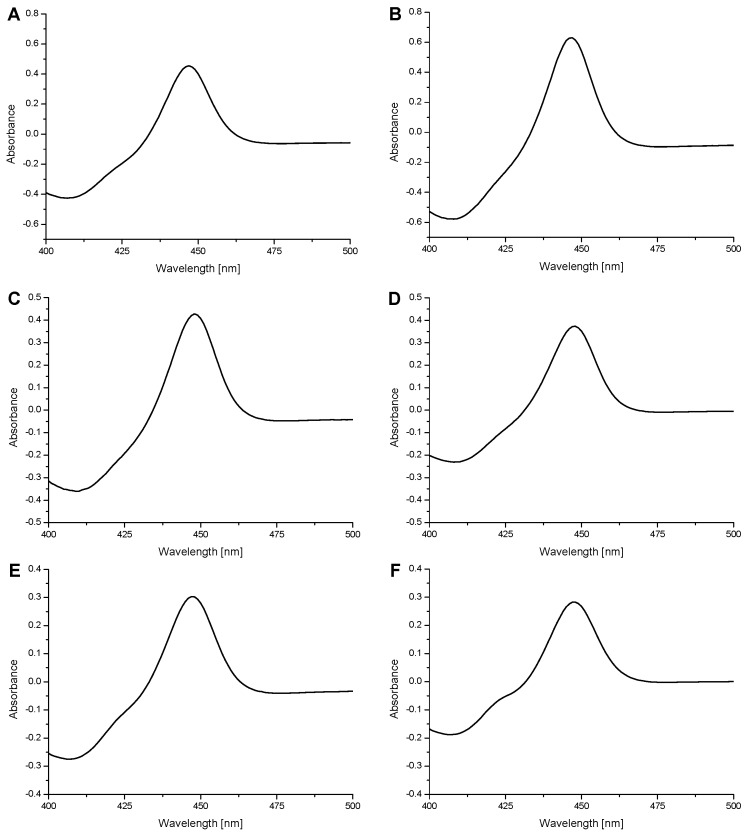
The CO-spectra of purified human CYP1A1 (**A**); its mutant S122A (**B**); CYP1A2 (**C**); its mutant T124V (**D**); CYP1B1 (**E**) and its mutant A133S (**F**). Fe^2+^-CO *versus* Fe^2+^ difference spectra.

The specific contents of individual CYPs were estimated to be 11.5, 11.6, and 9.4 nmol per mg protein for CYP1A1, 1A2, 1B1 enzymes, respectively, and 10.6, 8.7, and 9.9 nmol per mg protein for their mutants S122A, T124V, and A133S, respectively, based on the CO difference spectra (Figure 3) and the bicinchoninic acid colorimetric estimation method for total protein. The prepared enzymes were characterized using UV-vis spectroscopy to verify the presence of a correctly folded protein. The recorded CO-spectra of prepared human CYPs were essentially free of cytochrome P420 (Figure 3), indicating the correct fold and high quality of the prepared CYP enzymes.

POR, another enzyme of the monooxygenase system localized in the membrane of the endoplasmic reticulum, necessary for measuring the CYP-mediated enzymatic activity, was prepared by heterologous expression in *E. coli* as shown earlier [48]. In the case of POR, the rat enzyme was prepared and purified close to homogeneity (data not shown).

### 2.2. Examination of the Catalytic Activity of Human CYP1A1, 1A2 and 1B1 and Their Mutants to Oxidize Marker Substrates and AAI

**Figure 4:**
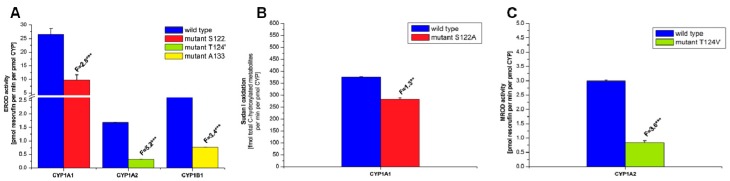
EROD (**A**), Sudan I oxidation (**B**) and MROD (**C**) catalyzed by human wild-type CYP1A1, 1A2 and 1B1, or their mutants CYP1A1-S122A, CYP1A2-T124V, and CYP1B1-A133S. All CYPs were reconstituted with rat POR in a ratio of 1:1 in liposomes. Data are averages ± SD of three independent measurements (*n* = 3). Numbers above columns (“F”) indicate fold changes in enzyme activity catalyzed by wild-type and mutant CYPs. Comparison was carried out by Student *t*-test; ** *p* < 0.01, *** *p* < 0.001, different from wild-type CYP enzymes.

In order to confirm the functional integrity of the prepared recombinant CYPs and their active site mutants, we investigated whether they are capable of catalyzing their marker activities, such as 7-ethoxyresorufin-*O-*deethylase (EROD), a marker activity for CYP1A1/2 and 1B1 [49], oxidation of Sudan I to its C-hydroxylation products, a marker for CYP1A1 activity [50], and 7-methoxyresorufin-*O*-demethylase (MROD) as a marker for CYP1A2 activity [49] (Figure 4). For these measurements and all other metabolism experiments all CYP enzymes were reconstituted with recombinant rat POR in liposomes. Although CYP1A1 is the most active enzyme of the CYP1 family for the EROD assay, other CYPs were also active to catalyze this reaction. All active site mutations in CYPs resulted in a significant decrease in EROD activity (up to 20%–40%) (Figure 4A). Similarly, oxidation of Sudan I catalyzed by CYP1A1 and its S122A mutant as well as MROD catalyzed by CYP1A2 and its T124V mutant were lower in the mutant forms than for the wild-type enzymes (Figure 4B,C). This indicates that the activity of CYP mutants was impaired in comparison to the wild-type form; however, they still show sufficient activity to demonstrate their ability to bind and metabolize marker substrates.

**Figure 5:**
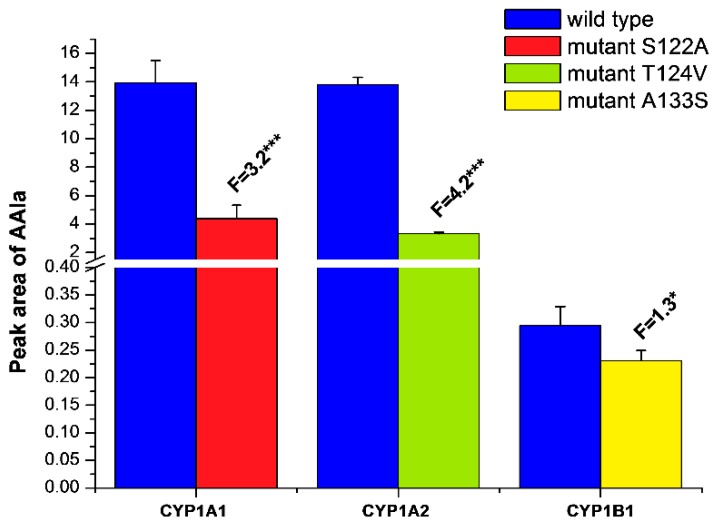
AAI *O*-demethylation to AAIa catalyzed by human wild-type CYP1A1, 1A2 and 1B1, and their mutants CYP1A1-S122A, CYP1A2-T124V, and CYP1B1-A133S. All CYPs were reconstituted with rat POR in a ratio of 1:1 in liposomes. Data are averages ± SD of three independent measurements (*n* = 3). Numbers above columns (“F”) indicate fold changes in *O*-demethylation of AAI to AAIa catalyzed by wild-type and mutant CYPs. Comparison was carried out by Student *t*-test; * *p* < 0.05, *** *p* < 0.001, different from wild-type CYP enzymes.

In previous studies we studied the activity of human CYP enzymes of the CYP1 family to *O*-demethylate AAI to its metabolite AAIa employing human recombinant enzymes heterologously expressed in microsomal fractions of baculovirus-infected insect cells in combination with POR (Supersomes™). We found that all three examined CYPs catalyzed this reaction [43,51]. In the present study we examined AAI oxidation using a more defined system, namely purified wild-type and mutant CYP1 enzymes in a reconstituted system with purified POR in liposomes. As shown in Figure 5, wild-type CYP1A1 and 1A2 reconstituted with POR exhibited essentially the same efficacy to oxidize AAI to AAIa, while wild-type CYP1B1 was more than 46-times less active in catalyzing this reaction. The AAI *O*-demethylation activity of CYP1A1 and CYP1A2 mutants decreased to approximately 25% of the corresponding wild-type forms; an analogous decrease was observed also for the marker substrates of CYP1A1/2 (Figure 4). Surprisingly, the AAI *O*-demethylation activity of the mutant A133S of CYP1B1 was decreased only negligibly (to ~80% of the wild-type) (Figure 5), which is in contrast to its large decrease in activity toward EROD. It is possible that the newly introduced serine residue stabilizes AAI in the active site, e.g., by H-bonding the AAI (see arrangement of the binary complex of the CYP1B1 active site with AAI shown in figure 7 of our previous study [38]), and thus partially compensates for the activity loss of CYP1B1 resulting from its mutation.

These results indicate that the prepared CYP mutants are enzymatically active in oxidative reactions but exhibit lower effectiveness. One explanation for this phenomenon could be that the active site mutations might change the active site structures dictating interactions with the test substrates and/or POR in the liposomal membrane, which finally lead to lower reaction rates. Moreover, the effect of the active site mutations on protein folding of the whole molecule of the mutant CYP proteins may also need to be considered. However, the latter suggestion is less probable because the prepared mutant forms of CYPs are essentially free of the misfolded protein form, cytochrome P420 (see Figure 3).

### 2.3. Examination of the Catalytic Activity of Human CYP1A1, 1A2 and 1B1 and Their Mutants to Reduce AAI to Species Forming AAI-DNA Adducts

**Figure 6:**
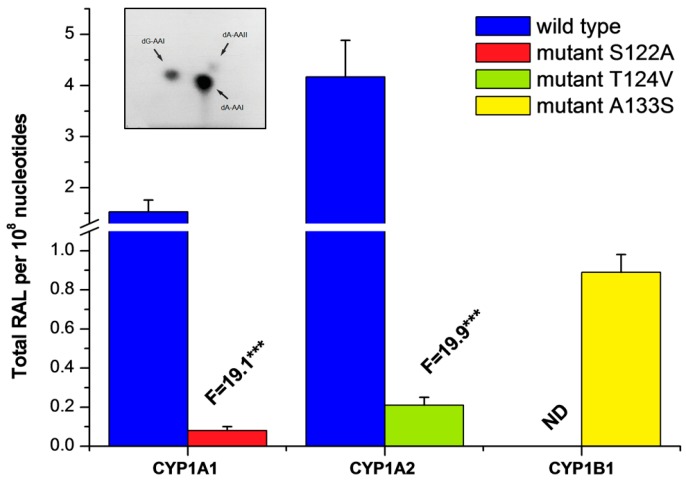
AAI-DNA adduct formation catalyzed by human wild-type CYP1A1, 1A2 and 1B1, and their mutants CYP1A1-S122A, CYP1A2-T124V, and CYP1B1-A133S reconstituted with rat POR in liposomes. Data are averages ± SD of three independent measurements (*n* = 3). The average amounts of adducts generated in the reference system containing only POR were subtracted, in order to eliminate the background related to the reduction not catalyzed by individual CYPs. RAL, relative adduct labelling. N.D.—not detected. Numbers above columns (“F”) indicate fold changes in AAI-DNA adduct formation catalyzed by wild-type and mutant CYPs. Comparison was carried out by Student *t*-test; *** *p* < 0.001, different from wild-type CYP enzymes. Insert: Autoradiographic profile of AAI-DNA adducts generated by reaction of AAI with wild-type CYP1A1 reconstituted with POR and DNA utilizing the nuclease P1 version of the ^32^P-postlabelling technique.

To investigate efficacy of the prepared human wild-type and mutant recombinant CYP1 enzymes in the reductive bioactivation of AAI to reactive intermediates that bind to DNA, they were reconstituted with rat recombinant POR in liposomes and utilized in reactions with AAI and DNA under anaerobic conditions. Liposomes containing only POR alone were used in incubations because the enzyme is known to be capable of reducing AAI [34,35,36,52]. The reconstituted systems containing human recombinant wild-type and mutant CYP1A1, 1A2, 1B1 plus POR or POR alone were all able to reduce AAI under anaerobic conditions. AAI-mediated DNA adduct formation determined by the ^32^P-postlabelling technique was generated by these enzymes (Table 1 and Figure 6). Three DNA adducts were formed by the enzymes (see adduct spots shown in the insert in Figure 6) and are the same as those found in renal DNA of patients suffering from AAN [16,53]. These adducts were previously identified to be the 7-(deoxyadenosin-*N*^6^-yl)aristolactam I (dA-AAI), 7-(deoxyguanosin-*N*^2^-yl)aristolactam I (dG-AAI) and 7-(deoxyadenosin-*N*^6^-yl)aristolactam II (dA-AAII) adducts (see insert in Figure 6). We have found in our previous work that the dA-AAII adduct may also be formed from AAI, probably *via* demethoxylation of AAI or dA-AAI [28,34,35,36]. POR-mediated AAI-DNA adduct formation was demonstrated to be dependent on the concentrations of POR (0.05–1.0 nmol POR in incubation mixture), and on the incubation time, being linear up to 0.6 nmol POR and 60 min, respectively (data not shown). Subsequently, 60 min incubations were carried out in the experiments where POR (0.2 nmol) was reconstituted with wild-type and mutant CYPs in liposomes in a ratio of 1:1. 

Higher levels—2.6- and 5.4-times—of AAI-DNA adducts were found when incubation mixtures contained, in addition to POR, wild-type CYP1A1 and 1A2, respectively, while no increase in generation of AAI-DNA adducts, in comparison to the reference containing only POR, was observed for human wild-type CYP1B1 (Table 1). When the CYP1A1-S122A and 1A2-T124V mutants were utilized, the formation of AAI-DNA adducts was almost abolished. In contrast, the reductive bioactivation of AAI to species forming DNA adducts was catalyzed by the A133S mutant of CYP1B1 compared with the wild-type form of this enzyme, adduct levels being ~2-fold higher than AAI activation catalyzed by POR alone (Table 1 and Figure 6).

**Table 1:** AAI-DNA adduct formation catalyzed by human wild-type CYP1A1, 1A2 and 1B1, and their mutants CYP1A1-S122A, CYP1A2-T124V, and CYP1B1-A133S. All CYPs were reconstituted with rat POR in a ratio of 1:1 or with rat recombinant POR alone in liposomes.

Enzymatic System	Levels of DNA Adducts in RAL *^a^* (Mean ± SD/10^8^ Nucleotides)			
	dG-AAI	dA-AAI	dA-AAII	Total
POR	0.22 ± 0.05	0.61 ± 0.08	0.12 ± 0.03	0.95 ± 0.14
CYP1A1 + POR	0.43 ± 0.08	1.89 ± 0.34	0.17 ± 0.04	2.49 ± 0.38 ***
CYP1A1-S122A mutant + POR	0.22 ± 0.06	0.71 ± 0.10	0.11 ± 0.05	1.04 ± 0.21
CYP1A2 + POR	0.89 ± 0.10	3.85 ± 0.48	0.38 ± 0.05	5.12 ± 0.87 ***
CYP1A2-T124V mutant + POR	0.24 ± 0.05	0.82 ± 0.10	0.10 ± 0.03	1.16 ± 0.23
CYP1B1 + POR	0.22 ± 0.05	0.61 ± 0.10	0.10 ± 0.05	0.93 ± 0.18
CYP1B1-A133S mutant + POR	0.40 ± 0.08	1.22 ± 0.14	0.21 ± 0.04	1.83 ± 0.19 ***

*^a^* RAL, relative adduct labelling; Comparison was carried out by Student *t*-test; *** *p* < 0.001, different from amounts of DNA adducts generated by POR alone.

These results indicate that the site-directed mutagenesis resulting in replacements of amino acid residues in the active centers of CYP1A1, 1A2 and 1B1 strongly influence AAI reductive bioactivation to species forming DNA adducts. Our results also confirmed the importance of the hydroxyl groups of amino acids in the CYP active center as donors of protons for AAI reduction as was previously predicted by theoretical studies [37,38]. The absence of the hydroxyl groups in Ala122 and Val124 in CYP1A1 and 1A2, respectively, resulted in a dramatic decrease in AAI nitroreduction activity, which consequently leads to negligible generation of AAI-DNA adducts. In contrast, the AAI-nitroreductase activity catalyzed by the CYP1B1 mutant containing Ser133 instead of Ala133 was clearly detected (Table 1, Figure 6). Therefore, these experimental observations confirm our theoretical prediction, which proposed that the presence of a hydroxyl group of amino acids in the CYP1A1/2 active site promotes the prominent nitroreduction of AAI [37,38].

## 3. Experimental Section

### 3.1. Vectors

The monocistronic plasmids with *CYP1A1* and *CYP1A2* genes were obtained courtesy of Professor Guengerich, F.P. (Vanderbilt University, Nashville, TN, USA). The pCWori+ expression plasmids contained the human *CYP1A1* and *CYP1A2* genes, which were modified for expression in *E. coli* as described [54,55,56] (Table 2). The *CYP1A1* gene was sequenced and an unexpected mutation was found in position I171L. Therefore, the mutated leucine was corrected to isoleucine by site-directed mutagenesis prior to further procedures. Both genes were modified to contain a C-terminal hexahistidine tag followed by a stop codon. To prepare this construct, the original genes were amplified with primers (Table 3) encoding a modified 3’-end of the coding sequence, digested by NdeI/HindIII and cloned back to a digested pCWori+ vector. 

The *CYP1B1* gene cloned in pOTB7 was obtained from the Harvard PlasmID Database (clone MGC:19842; allele CYP1B1*3). cDNA was amplified with primers encoding the 5’-end terminal modification [57] (Table 2) and 3’-end hexahistidine tag. The fragment was digested with NdeI/HindIII and ligated into the pCWori+ plasmid. Site-directed mutagenesis was used to correct the L432V mutation to yield the wild-type enzyme [58].

**Table 2:** N-terminal modifications of CYPs used in this study.

**CYP1A1**	WT	MLFPISMSATEFLLASVIFCLVFWVM
	Modified	MAFPISMSATEFLLASVIFCLVFWVM
**CYP1A2**	WT	MALSQSVPFSATELLLASAIFCLVFW
	Modified	MA------------LLAVFLFCLVFW
**CYP1B1**	WT	MGTSLSPNDPWPLN
	Modified	M---LSPNDPWPLN

The plasmid pETOR262 with rat POR was a gift of Professor Szklarz, G.D. (West Virginia University, Morgantown, WV, USA). Plasmid pETOR262 [59] included the *POR* gene with N-terminal ompAIII fusion [48].

### 3.2. Construction of CYP Mutants

Mutant forms of CYP1A1, CYP1A2 and CYP1B1 were prepared using Quickchange Lightning Site-directed Mutagenesis kit for CYP1A1 and 1B1 and Quickchange II XL Site-directed Mutagenesis kit for CYP1A2 (Agilent Technologies, Santa Clara, CA, USA). Plasmids with wild-type genes were used as templates and primers (Table 3) were designed according to the manufacturer’s manual.

**Table 3:** Primers used for *CYP* gene modifications.

**1A1**	Correction of I171L
	Forward GAGGCTGAGGTCCTGATAAGCACGTTGCAGG
	Reverse CCTGCAACGTGCTTATCAGGACCTCAGCCTC
**1a1**	Histidine tag modification
	Forward GAATTCATATGGCTTTTCCAATTTCAATG
	Reverse TATCTAAGCTTCATTAATGATGATGATGATGATGAGAGCGCAGCTGCATTTG
**1A1**	S122A mutation
	Forward GTAATGGTCAGAGCATGGCCTTCAGCCCAGACTC
	Reverse GAGTCTGGGCTGAAGGCCATGCTCTGACCATTAC
**1A2**	Histidine tag modification
	Forward GAATTCCATATGGCTCTGTTATTAGCAG
	Reverse TATCTAAGCTTCATTAATGATGATGATGATGATGATTGATGGAGAAGCGCAG
**1A2**	T124V mutation
	Forward CTGGCCAGAGCTTGGTCTTCAGCACAGACTCTG
	Reverse CAGAGTCTGTGCTGAAGACCAAGCTCTGGCCAG
**1B1**	N-terminal and histidine tag modification
	Forward GACGAATTCATATGCTTTCTCCAAATGATCCATGGCCGCTAAACCCG
	Reverse GCCAAGGAAACTTGCCAACATCATCATCATCATCATTAATGAAGCTTAGATA
**1B1**	Correction of L432V
	Forward CTGTGAATCATGACCCACTGAAGTGGCCTAACCCG
	Reverse CGGGTTAGGCCACTTCAGTGGGTCATGATTCACAG
**1B1**	A133S mutation
	Forward CGGCGGCCGCAGCATGTCTTTCGGCCACTACTC
	Reverse GAGTAGTGGCCGAAAGACATGCTGCGGCCGCCG

### 3.3. Expression of Human CYPs and Their Purification

Competent *E. coli* JM109 cells (Promega, Corp., Madison, USA, genotype [*endA1 glnV44 thi-1 relA1 gyrA96 recA1 mcrB^+^ Δ(lac-proAB) e14- [F' traD36 proAB^+^ lacI^q^ lacZΔM15*] hsdR17(r_K_^−^m_K_^+^)]) were transformed with each of the expression plasmids and plated on Luria Broth (LB) agar containing 100 mg ampicillin/L overnight. 

All chemicals used in additional experiments were from Sigma Chemical Co (St. Louis, MO, USA). A single isolated colony was picked and the starting culture was cultivated overnight in a LB medium with 100 mg ampicillin/L at 37 °C at 220 rotations per minute (RPM). The starting culture was then diluted 1:100 in a modified Terrific Broth (TB) medium (12 g tryptone, 24 g yeast extract, 2 g bactopeptone and 4 mL of glycerol) with 100 mg ampicillin/L and 1 mM thiamine and supplemented with minerals (0.25 mL of stock preparation per liter of culture; composition: 24.5 g ferric citrate, 1.31 g ZnCl_2_, 2 g CoCl_2_ × 6H_2_O, 2 g Na_2_MoO_4_ × 2H_2_O, 1 g CaCl_2_ × 2H_2_O, 1.27 g CuCl_2_ × 2H_2_O, 0.5 g H_3_BO_3_ and 100 mL concentrated HCl per liter). Bacteria were grown at 37 °C at 220 RPM. Induction of CYP expression was initiated by the addition of 1 mM isopropyl β-d-1-thiogalactopyranoside (IPTG), when the optical density at 600 nm was approximately 0.8. Addition of δ-aminolevulinic acid to reach its concentration of 0.5 mM for CYP1A1 and CYP1B1 was also done at this time. The induced culture was shaken vigorously for 48 h at 30 °C.

Bacterial membranes were essentially prepared as described [55]. All subsequent experiments were performed at 4 °C. Cells were harvested by centrifugation at 4000× *g* for 15 min and resuspended in 100 mM Tris-acetate buffer pH 7.6 containing 500 mM sucrose and 0.5 mM EDTA (5 mL per 100 mL of the culture). After adding of lysozyme (0.2 mg/mL), the cells were shaken for 30 min and the mixture was diluted twofold with cold distilled water. The obtained spheroplasts were centrifuged at 4000× *g* for 15 min and the pellet was resuspended (4 mL per 100 mL of the culture) in 100 mM potassium phosphate buffer (pH 7.4) containing 6 mM magnesium acetate, 20% glycerol (*v*/*v*) and 5 mM β-mercaptoethanol. The spheroplasts were stored at −70 °C until further use.

The protease inhibitors aprotinin, leupeptin, bestatin and phenylmethylsulfonyl fluoride (PMSF) (in 2-propanol) were added to reach their concentrations of 1 mg/mL, 2 μM, 1 μM and 1 mM, respectively. Spheroplasts were sonicated six times for 30 s each, on ice, and centrifuged at 10,000× *g* for 20 min. Supernatants were centrifuged at 180,000× *g* for 60 min. The obtained membranes were resuspended (100 mL per liter of culture) in 100 mM potassium phosphate buffer (pH 7.4) containing 500 mM sodium chloride, 1% CHAPS, 20% glycerol (*v*/*v*), 5 mM β-mercaptoethanol, 30 μM α-naphthoflavone and stirred for 3 h. The mixture was then centrifuged at 100,000× *g* for 60 min to discard any insoluble material.

The supernatant was then applied on a column of Ni-NTA agarose (QIAGEN, Hilden, Germany) equilibrated with the 100 mM potassium phosphate buffer (pH 7.4) containing 500 mM sodium chloride, 0.5% CHAPS, 20% glycerol (*v*/*v*), 5 mM β-mercaptoethanol, and 30 μM α-naphthoflavone. The bound protein was washed extensively with 100 mM potassium phosphate buffer (pH 7.4) containing 500 mM sodium chloride, 0.5% CHAPS, 20% glycerol (*v*/*v*), and 30 mM imidazole. The CYP proteins were eluted with 100 mM potassium phosphate buffer (pH 7.4) containing 500 mM sodium chloride, 0.5% CHAPS, 20% glycerol (*v*/*v*), and 300 mM imidazole. The detergent was removed by triple dialysis against 200-fold volume of 100 mM potassium phosphate buffer (pH 7.4) containing 20% glycerol (*v*/*v*). Protein was concentrated using Amicon centrifugal filter units and dialyzed against the same buffer.

### 3.4. Rat POR Expression and Purification

Plasmid pETOR262 was transformed into *E. coli* strain JM109(DE3) (Promega, Corp., Madison, WI, USA, genotype [*endA1 glnV44 thi-1 relA1 gyrA96 recA1 mcrB^+^ Δ(lac-proAB) e14-[F' traD36 proAB^+^ lacI^q^ lacZΔM15] hsdR17(r_K_^−^m_K_^+^) λ(DE3)*]). Cells were cultivated at 37 °C in LB broth at 220 RPM containing 1 μg/mL riboflavin and 50 μg/mL kanamycin to an *A*_600_ of approximately 0.8 and then induced with 0.1 mM isopropyl β-d-1-thiogalactopyranoside (IPTG). The cells were then cultivated for further 20 h at 30 °C at 190 RPM.

Cultures were harvested by centrifugation at 4000× *g* for 15 min at 4 °C and pelleted cells were resuspended in 75 mM Tris-Cl (pH 8) containing 250 mM sucrose, 0.25 mM EDTA, 0.02 mg/mL lysozyme using ratio of 80 mL buffer to 1 liter of original culture. Resuspended cells were incubated on ice for 30 min with gentle shaking. Prepared spheroplasts were centrifuged at 4000× *g* for 15 min at 4 °C, resuspended in 50 mM Tris-Cl (pH 8), 0.5 mM EDTA and sonicated 6× for 30 s each, on ice, and centrifuged at 180,000× *g* at 4 °C for 60 min.

The obtained membranes were resuspended in buffer A (50 mM Tris-Cl (pH 8) containing 0.1 mM EDTA, 0.05 mM dithiotreitol (DTT), 10% glycerol (*v*/*v*) and 0.1% Triton X-100 (*v*/*v*)) and incubated 3 h at 4 °C. The mixture was centrifuged at 100,000× *g* at 4 °C for 60 min to remove unsolubilized material and supernatant was applied to a 2’,5’-ADP Sepharose 4B affinity column (1 × 5 cm) equilibrated with a buffer A. The bound sample was washed with 100 mL of buffer A and finally eluted with buffer A containing 2 mM 2-AMP. Triton X-100 was eliminated by application of the eluate directly on a small DEAE-Sepharose column (1 × 3 cm) and washing the bound protein with 20 column volumes of 10 mM Tris-Cl (pH 8) containing 20% glycerol, 0.1 mM EDTA, 0.05 mM DTT [47]. Protein was eluted by same buffer with 400 mM sodium chloride, concentrated using Amicon centrifugal filter units, dialyzed against 30 mM potassium phosphate (pH 7.4), 0.5 mM EDTA, 20% glycerol, 2 μM flavine mono nucleotide (FMN) overnight and then against same buffer without addition of FMN.

### 3.5. Determination of CYP and Protein Contents

The concentration of CYP was determined as described by Omura and Sato [60] based on the absorption of the complex of reduced CYP with carbon monoxide. Protein concentrations were determined using a bicinchonic acid assay (BCA, Thermo Fisher Scientific, Waltham, MA, USA) with bovine serum albumin as a standard [61].

### 3.6. Measurement of CYP1A1-, 1A2- and 1B1-Mediated EROD Activities

Human recombinant CYPs reconstituted with rat POR were analyzed for specific CYP1A and 1B1 activities by measuring 7-ethoxyresorufin *O*-deethylation (EROD) as described by Burke and Mayer [49]. Briefly, incubation mixtures (150 μL) contained 100 mM potassium phosphate buffer (pH 7.4), 0.5 mM NADPH, 50 nM CYPs reconstituted with POR (in a ratio of 1:1) in liposomes and 2.2 μM 7-ethoxyresorufin (dissolved in 1.5 μL dimethyl sulfoxide, DMSO) [62]. The reaction was starting by addition of NADPH. CYPs were reconstituted with POR as follows: 200 pmol CYP with 200 pmol POR, 0.1 mM liposomes prepared from dilauroyl phosphatidylcholine, 3 mM reduced glutathione, and 50 mM HEPES/KOH buffer, pH 7.4 [34,63]. Human CYPs reconstituted with rat recombinant POR form the enzymatic systems analogous to those with human POR [50,63]. The formation of resorufin was measured on a luminescence spectrometer (PerkinElmer LS-55 equipped with 96-well plate reader) for 10 min at ambient temperature by measuring its fluorescence (excitation and emission wavelengths of 530 and 585 nm, respectively) [62]. The rate of dealkylation was evaluated on the basis of a resorufin standard curve.

### 3.7. Measurement of CYP1A1-Mediated Oxidation of Sudan I

Human recombinant CYP1A1 and its mutant S122A reconstituted with POR were analyzed for specific CYP1A1 activity by measuring of the oxidation of Sudan I (1-phenylazo-2-naphthol), as described previously [50]. Incubation mixtures used to study Sudan I oxidation had a final volume of 500 μL and contained 50 mM potassium phosphate buffer (pH 7.4), 1 mM NADPH, 50 nM CYP1A1 or its mutant S122A reconstituted with POR (in a ratio of 1:1) in liposomes and 0.1 mM Sudan I (dissolved in 5 μL methanol). CYPs were reconstituted with POR as described above. The reaction was started by addition of NADPH. After incubation (37 °C, 30 min), Sudan I metabolites were extracted with ethyl acetate (2 × 1 mL), the extracts evaporated and residues dissolved in 25 μL methanol. Oxidation of Sudan I by CYP1A1 enzymes has been shown to be linear up to 30 min [50]. HPLC analysis was performed as described [50]. 

### 3.8. Measurement of CYP1A2-Mediated MROD Activity

Human recombinant CYP1A2 and its mutant T124V reconstituted with POR were analyzed for specific CYP1A2 enzyme activity by monitoring MROD activity as described [49]. Briefly, incubation mixtures (150 μL) contained 100 mM potassium phosphate buffer (pH 7.4), 0.5 mM NADPH, 50 nM CYP1A2 or its mutant T124V reconstituted with POR (in a ratio of 1:1) in liposomes and 2.2 μM 7-methoxyresorufin (dissolved in 1.5 μL dimethyl sulfoxide, DMSO). The reaction was started by addition of NADPH. CYPs were reconstituted with POR as described above. The formation of resorufin was measured as described above.

### 3.9. Incubations to Study AAI Oxidation to AAIa by Human Recombinant CYPs

Incubation mixtures (500 μL) used for determining the *O*-demethylation of AAI (sodium salt, ≥97%, Sigma-Aldrich, St. Louis, MO, USA) to AAIa catalyzed by CYPs reconstituted with POR in liposomes contained 100 mM potassium phosphate buffer (pH 7.4), 1 mM NADPH, 50 nM CYPs reconstituted with POR (in a ratio of 1:1, see above) in liposomes and 10 μM AAI (as sodium salt dissolved in water). The reaction was started by addition of NADPH. AAI and AAIa were extracted from incubation mixtures with 2 × 1 mL of ethyl acetate and evaporated to dryness. Residues were dissolved in 30 μL of methanol and analyzed by reverse-phase HPLC as described [39,51].

### 3.10. Determination of AAI-DNA Adduct Formation by ^32^P-Postlabelling

The deaerated and nitrogen-purged incubation mixtures (750 μL) contained 50 mM potassium phosphate buffer (pH 7.4), 1 mM NADPH, 0.2 nmol CYPs reconstituted with 0.2 nmol POR in liposomes, 0.5 mM AAI (as sodium salt dissolved in water) and 1 mg of calf thymus DNA (4 mM). The reaction was started by addition of NADPH. Incubations were performed at 37 °C for 60 min. Control incubations were performed either without activating system (CYPs with POR in liposomes) or without CYPs in liposomes (POR alone was present in liposomes) or with activating system and AAI, but without DNA or with activating system and DNA but without AAI. Reconstitution of human recombinant CYP1A1, 1A2 and 1B1 and their mutants with rat recombinant POR was carried out as described above. In incubations testing the activity of rat recombinant POR the incubation mixtures were essentially the same as in the reconstitution experiments, except that the CYPs were omitted from the reconstitution mixture. After incubation (37 °C, 60 min), all reaction mixtures were extracted with ethyl acetate (2 × 2 mL). DNA was isolated from the residual water phase by a standard phenol/chloroform extraction method. The ^32^P-postlabelling technique using the nuclease-P1 version and thin-layer chromatography (TLC) for the analysis of AAI-DNA adduct formation was carried out as shown previously [34]. TLC sheets were scanned with a Packard Instant Imager (Dowers Grove, IL, USA) and DNA adduct levels (RAL, relative adduct labeling), where RAL were calculated as RAL = cpm in adduct/cpm in total (normal) nucleotides as described [16,43,44,52]. Results were expressed as DNA adducts/10^8^ nucleotides. 

### 3.11. Statistical Analyses

Statistical analyses were carried out with means ± standard deviations of three parallel experiments with Student’s *t*-test (UNISTAT Statistics Softwere v6, Unistat Ltd., Highgate, London N6 5UQ, UK) and *p* < 0.05 was considered significant. 

## 4. Conclusions

The results found in the present work confirm our previous studies demonstrating that the human carcinogen AAI is, under anaerobic conditions, reductively activated by human CYP1A1 and 1A2 to species forming AAI-DNA adducts [34,38,52] and contribute to the explanation, why just these CYPs, and not closely related CYP1B1, catalyze this reaction. This is an important feature, mainly because of the fact that the mechanism responsible for CYP-catalyzed nitroreduction is still not fully resolved [37,38,64]. 

Even though the results found in our studies utilizing theoretical methods [37,38] indicated analogous binding of AAI to the active centers of CYP1A1, 1A2 and 1B1, some differences in orientations of AAI in the AAI-CYP1A1/2 and AAI-CYP1B1 binary complexes occur. The AAI molecule is bound in such a way as to inhibit binding of O_2_. Indeed, when AAI is bound to the active sites of all three CYPs, its carboxylic group is located directly above their heme iron. This ligand orientation in CYP1A1 and 1A2 is additionally stabilized by two hydrogen bonds; one between an oxygen atom of the nitro group of AAI and the hydroxyl group of Ser122 and Thr124 of CYP1A1 and 1A2, respectively, and the second bond between an oxygen atom of the dioxolane ring of AAI and the hydroxyl group of Thr497 and Thr498 of these CYPs [37,38]. The hydrogen bond between an oxygen atom of the nitro group of AAI and the hydroxyl group of the amino acid residues cannot, however, occur in the binary complex of CYP1B1 with AAI; no amino acids containing the hydroxyl group are present in the CYP1B1 active center. The hydroxyl group containing amino acid residues are replaced by the hydrophobic amino acid Ala133 in CYP1B1 [37,38]. Hence, these studies suggested that the hydroxyl group of amino acids Ser122/Thr124 is mechanistically important, providing a proton required for the reduction of AAI [37,38]. In the present experimental work, the site-directed mutagenesis was utilized as a tool to replace the above mentioned amino acids in the active centers of CYP1A1, 1A2 and 1B1 and the prepared mutants were utilized for the experimental study. The S122A/T124V mutations in CYP1A1/1A2 nearly completely obliterated the nitro-reduction of AAI to form AAI-DNA adducts. On the contrary, the AAI reduction was established by the A133S mutation in CYP1B1. These findings confirm the importance of the hydroxyl group containing amino acid residues in the active sites of the examined CYPs for their potency to catalyze the reduction of AAI. Moreover, the results of this and previous studies [37,38] indicate that the combination of experimental approaches with the theoretical prediction is an important step to explain the mechanism of the CYP-mediated reductive reactions.
